# Effects of air pollution on cardiovascular health in patients with type 2 diabetes mellitus: Evidence from a large tertiary hospital in Shandong Province, China

**DOI:** 10.3389/fpubh.2022.1050676

**Published:** 2022-11-09

**Authors:** Jitian Zhang, Dong Ren, Shuo Wang, Sha Zhu, Kai Qu, Yuan Yuan

**Affiliations:** ^1^Clinical Nutrition Department, Shandong Provincial Hospital Affiliated to Shandong First Medical University, Jinan, China; ^2^Scientific Research Management Department, Shandong Academy of Macroeconomic Research, Jinan, China; ^3^The Center for Economic Research, Shandong University, Jinan, China; ^4^Medical Administration Department, Shandong Provincial Hospital Affiliated to Shandong First Medical University, Jinan, China; ^5^Shandong Provincial Eco-environment Monitoring Center, Jinan, China

**Keywords:** air pollution, type 2 diabetes mellitus, cardiovascular health, group differences, instrumental variables regression

## Abstract

Air pollution has posed serious threats to human health. Based on the microdata of a large tertiary hospital in Shandong Province from 2016 to 2021, combined with the macro data such as air quality monitoring data, meteorological data, and city-level regional socio-economic data, this paper empirically tests the impact of air pollution instrumented by thermal inversions on the cardiovascular health of patients with type 2 diabetes mellitus (T2DM) and its group differences. The results show that: (1) Air pollution has a negative impact on the cardiovascular health of patients with T2DM, that is, the cardiovascular health of patients with T2DM will decline in regions with high air pollution; (2) The impact of air pollution on cardiovascular health in T2DM patients is heterogeneous, with males and older patients bearing greater air pollution health losses; (3) From the perspective of the external environment, the negative effects of environmental pollution on patients' health were significantly reduced in areas with higher environmental regulation intensity and better public health conditions, indicating the necessity of strengthening environmental governance and increasing public health expenditure.

## Introduction

With the development of the economy and the change in environment and lifestyle, the burden of diabetes continues to increase worldwide. The global prevalence of diabetes had reached pandemic proportions with a prevalence of 10.5% (537 million adults) in 2021, and the number of adults with diabetes will continue to rise rapidly and is expected to reach 643 million by 2030 and 783 million by 2045 (1). Cardiovascular disease (CVD) is a major complication in patients with T2DM. Studies have revealed that approximately one in three adults with T2DM had established CVD, and nearly half of all deaths in patients with diabetes could be attributed to CVD (2, 3). Therefore, cardiovascular complications of T2DM have caused serious harm to patients, families, and society.

In recent years, with the acceleration of global industrialization and urbanization, air pollution has become an increasingly prominent problem, which has attracted great attention all over the world. In particular, fine particulate pollution from automobile engines, fireplaces, and coal-fired power plants is easily inhaled, easily enriched with toxic and harmful substances, and widely dispersed. Thus, air pollution affects nearly every organ in the body, causing or contributing to many diseases, such as respiratory diseases, cardiovascular diseases, and even death. The World Health Organization (WHO) warns that all people on the planet are exposed to air pollution levels. The Global Burden of Disease (GBD) study shows that air pollution is the fourth leading risk factor for human mortality. Air pollution is responsible for 9 million deaths worldwide, of which 61.9% are due to CVDs, including ischaemic heart disease (31.7%) and stroke (27.7%) (4). Air pollution is a challenge facing nearly every country in the world. No country, rich or poor, can be immune to air pollution.

Similarly, poor air quality is considered as a major issue in China. Yin et al. (5) estimated the effect of air pollution on deaths, disease burden, and life expectancy across China and its provinces from 1990 to 2017, and found that air pollution remains an important risk factor in China, with 81% living in regions exceeding the WHO Interim Target 1, and 1.24 million deaths in China were attributable to air pollution in 2017, 40.0% of disability-adjusted life-years (DALYs) for chronic obstructive pulmonary disease (COPD) were attributable to air pollution, as were 19.5% for ischaemic heart disease, and 12.8% for stroke. In recent years, the Chinese government has introduced policies, such as the “Action Plan of Air Pollution Prevention and Control,” aiming to improve the environment and promote the transformation of the mode of social and economic development. However, does air pollution have a significant impact on cardiovascular health in patients with T2DM? Few scholars have conducted detailed studies. If yes, are there any group differences among different genders and ages? Can government regulation and public health expenditure mitigate or even curb the negative health effects of air pollution? Therefore, it is necessary to quantify the environmental costs of economic policies and to rigorously assess the potential risks to cardiovascular health in patients with T2DM.

The study on the health effects of environmental pollution begins with the health production function proposed by Grossman (6), which describes the relationship between health input and health output. Improving living conditions and sanitation and creating a good environment are considered to be important sources of increasing the health capital stock. This function has become the theoretical basis for mainstream health research in the field of economics. Since then, many scholars have incorporated environmental factors into the health production function and investigated the impact of environmental factors on the health depreciation rate (7–9). Most of the existing studies focus on the field of medicine and public health. Based on micro-individual or non-random samples, epidemiological methods are used to study the dose-response relationship between the concentration of air pollutants and the health of exposed people, and further analyze the impact of air pollution on the health of residents' respiratory system, cardiovascular and cerebrovascular system. For example, a prospective epidemiological study by Hystad et al. (10), based on 157,436 adults aged 35–70 years from 747 communities in 21 high-income, middle-income, and low-income countries, found that an increase of 10 μg/m ^3^ in particulate matter less than or equal to 2.5 mm in aerodynamic diameter (PM2.5), cardiovascular events increase by 5%, myocardial infarction increase by 3%, stroke increase by 7%, and CVD mortality increase by 3%. There are few studies on the health effects of air pollution from the perspective of economics. Scholars tend to use quasi-natural experiments to solve the endogenous disturbances of environment and health and evaluate the effects of the environment on health through econometric models. Bayat et al. (11) estimated PM2.5 exposure in 349 communities in Tehran through the Environmental Benefits Mapping and Analysis Program (BenMAP-CE) and used the Global Exposure Mortality Model (GEMM) to estimate the avoidable burden attributable to ambient air pollution in Tehran; the economic and health effects of changes in PM2.5 concentrations were estimated using the value of life-year (VOLY) method. Palma et al. (12) analyzed the causal effect of prenatal exposure to air pollution on neonatal health in Italy in the 2000s by combining detailed information on the mother's residential location from birth certificates with particulate matter less than or equal to 10 mm in aerodynamic diameter (PM10) concentrations from air pollution monitors, and found that an average increase of 10 units of PM10 level would decrease birth weight by about 0.5% and the gestational age by 0.16%; it would increase the prevalence of low birth weight by 22% and of preterm birth by 16%. Maji et al. (13) analyzed the seasonal variation of ground-level O_3_ in 338 cities in China during the year 2016 and assessed the city-specific cardiovascular and respiratory disease-related mortality attributable to ozone exposure by the log-linear and linear model, and estimated the city-specific total economic loss in China by the log-linear model. Gu et al. (14) investigated the relationship between air pollution and residents' health by nesting the household registration data of the China Migrant Dynamic Survey in 2014 with city characteristic data and pollution data, and found that the increase in air pollution concentration significantly reduced the health level of residents; after solving the endogenous problems with the instrumental variable (IV)-the ordered probit (OProbit) model, the negative impact of air pollution on residents' health remained significant.

As mentioned above, extensive medical and economic evidence have proved the negative health impacts of exposure to air pollution. Thus, whether environmental regulation and public health services can be used as a breakthrough to recover residents' health losses is a new topic of concern for scholars. Given the short-term effects of environmental policies on health, scholars mainly discuss the effects on health in a relatively short period before and after the implementation of temporary policies. For example, in 2004, the Republic of Ireland implemented a national smoking ban in the workplace. Following the ban implementation, there was a 13% decrease in all-cause mortality, a 26% decrease in ischemic heart disease, a 32% decrease in stroke, and a 38% decrease in COPD within a few weeks (15). For the 2008 Beijing Olympics, the Chinese government imposed factory emissions and travel restrictions from July 1 to September 20. During the implementation period, air pollutant concentrations in Beijing decreased by 62%, resulting in a range of health benefits, including improved lung function in healthy adults and asthmatic adults, a 58% reduction in asthma-related physician visits, lower CVD mortality in women and the elderly, and lower degrees of systemic inflammation among healthy young adults (16). However, the impact of some environmental policies or measures may be lagged after implementation. The implementation of current air pollution prevention policies sometimes has little impact on the environmental quality of the current period, but a large impact on the environmental quality of the next period. Therefore, scholars pay more attention to the long-term health effects of environmental policies. The Clean Air Act, enacted in 1970 and further amended in 1977 and 1990, is the most influential environmental regulation in U.S. history. Since 1990 there has been approximately a 50% decline in emissions of key air pollutants, which also brought dramatic health benefits, 230,000 deaths per year averted from lower concentrations of outdoor PM, 7,100 premature deaths per year averted from lower ozone concentrations, 200,000 fewer acute myocardial infarction cases per year, and 2.4 million asthma exacerbation are prevented each year (17–19). In Western Europe and the European Union, air quality regulations have significantly reduced air pollution and improved small-airway health in adults. Schindler et al. (20) found that from 1991 to 2002, PM10 in Switzerland decreased by 6.2 mg /m^3^ on average and was associated with 259 fewer people with regular cough, 179 fewer people with chronic cough or sputum, and 137 fewer people with dyspnea or shortness of breath per 10,000 people in the community. In China, the research on the long-term health effects of environmental policies has just started, and most of the relevant studies are qualitative discussions with relatively little empirical evidence.

To date, health assessment studies on environmental policies are mainly conducted in foreign countries. Most of the studies on the health effects of air pollution in China focus on the fields of medicine and public health and are mainly characterized by statistics. Few studies discuss the health effects of environmental policies from the perspective of economics. To this end, based on the data of T2DM patients from Yi Du Cloud Medical Research Data Platform of Shandong Provincial Hospital Affiliated to Shandong First Medical University, this paper incorporates environmental factors into the health production function to discuss the impact of air pollution on the cardiovascular health of T2DM patients. The main contributions of this paper are as follows. Firstly, compared with medical and public health research, this paper analyzes the effects of air pollution on health from micro and macro dimensions. Secondly, this paper focuses on the effects of air pollution on cardiovascular health in patients with T2DM, which is different from previous studies on the general population. Thirdly, this paper further explores the differential effects of air pollution on cardiovascular effects in T2DM patients of different genders and ages and conducts heterogeneity analysis based on environmental regulations and medical health conditions, which has theoretical and practical implications.

## Theoretical background and hypotheses

### The effect of air pollution on cardiovascular health in patients with T2DM

In 2021, WHO updated Global Air Quality Guidelines, pointing out that no country met the latest WHO PM2.5 air quality guidelines, suggesting that air pollution poses a major threat to health, and calling on all countries in the world to adopt a “health in all policy” to solve the pollution problem. Theoretically, air pollution can damage the function of the respiratory and circulatory systems and have an obvious negative impact on human health. A large number of medical and epidemiological studies have shown that air pollution (such as sulfur dioxide, atmospheric particulate matter, etc.) is the cause of CVDs in residents (21, 22). However, only a part of the health consequences of environmental pollution can be observed, and current studies have not provided sufficient evidence of the magnitude of the negative health effects. To explore the influence of environmental factors on the occurrence and development of T2DM, this paper included environmental factors in the research scope of health economics to evaluate the effect of air pollution on cardiovascular health in patients with T2DM, which would be the first theoretical hypothesis to be tested in this study:

#### Hypothesis 1

Air pollution has a negative effect on cardiovascular health in patients with T2DM, controlling for other confounding factors.

### The population difference in the effects of air pollution on cardiovascular health in patients with T2DM

Based on the environmental equity theory, environmental pollution and environmental risk exposure have different effects on the health of different social groups. To a large extent, air pollution can have an impact on human cardiovascular health through other systems such as the respiratory system. However, individual differences (characteristics, geographical, economic development level, etc.) can cause environmental iniquity, whether in the same environment or not, the effects of health on groups with different individual characteristics may show heterogeneity. In 2020, China's seventh national census showed that the number of people aged 60 and above reached 264 million, accounting for 18.7 percent of the total population, an increase of 5.44% compared with 2010, indicating a further deepening of population aging. In addition, the worse the air quality, the greater the risk of disease in the elderly, and the lower the health level of the elderly. Honda et al. (23) studied 4,121 people over 57 years old in the United States and found that air pollution may be a key risk factor for abnormal glucose metabolism and diabetes in the elderly. Then, is there also age heterogeneity in the impact of air pollution on cardiovascular health, the main complication in patients with T2DM? Similarly, compared with women, men are more likely to engage in outdoor physical activities and are exposed to air pollution for a longer time, is there gender heterogeneity in the impact of air pollution on cardiovascular health in patients with T2DM? Thus, this paper puts forward Hypothesis2 (H2):

#### Hypothesis 2

The effects of air pollution on cardiovascular health in patients with T2DM show gender and age differences: male and the elderly bear more environmental losses and greater health risks than female and the young.

### The effect of environmental regulation and public health input on health effects of pollution

The externality of environmental public goods may lead to differences in the cost of health damage caused by air pollution among different groups, but previous empirical studies in the field of economics rarely consider this difference. Yang and Zhang (24) estimated the impact of air pollution exposure on household healthcare expenditure by using the China Urban Household Survey (UHS) Database, and showed that a 1% increase in yearly exposure to PM2.5 would lead to a 2.94% increase in household health expenditure. It is estimated that the 13th Five-Year Plan for Ecological and Environmental Protection will reduce annual national medical and health expenditures by 47.36 billion U.S. dollars, accounting for 0.64% of China's gross domestic product (GDP). Giaccherini et al. (25) analyzed the heterogeneous effects of particle pollution on Italian daily hospitalizations and their costs and found that a one standard deviation increase in PM10 leads to additional 0.79 hospitalizations per 100,000 residents, total daily costs represent 0.5% of the total daily health expenditure. However, as an important environmental public good, can strengthening environmental regulation and public health investment reduce the health harm caused by air pollution? This perspective is rarely involved in domestic research. Economic development is bound to damage and affect the environment, and environmental pollution is an important factor affecting people's health. However, economic development also allows the government to invest more funds in public health to improve people's livelihood and health. Therefore, should we follow the old path of “development first, governance later”? Or should we strengthen environmental regulation within the limits of the self-purification of the ecological environment, to minimize the damage to the environment in the process of economic development, and at the same time, we make good use of the economic dividends and increase the investment in public health? In view of this, this paper proposes the following assumptions:

#### Hypothesis 3

In areas with enhanced environmental regulation and increased public health investment, patients enjoy higher environmental welfare and bear smaller health risks.

## Materials and methods

### Data sources and processing

In this paper, we combine the microdata of patients with T2DM with the macro data such as air quality monitoring data, meteorological data, and city-level regional socio-economic data, to evaluate the impact of air pollution on the cardiovascular health of patients with T2DM.

Firstly, the health data comes from the Yi Du Cloud Medical Research Data Platform, which collects the data of patients with T2DM admitted to the Endocrinology Department of Shandong Provincial Hospital Affiliated to Shandong First Medical University from 2016 to 2021, including basic information such as age and gender, health behavior information such as smoking, alcoholism, and various medical laboratory indicators, and the prevalence of CVDs, which is considered by scholars to be very detailed data for studying health problems. After matching, a total of 1,446 T2DM patients were enrolled, covering 16 prefecture-level cities in Shandong Province.

Secondly, the air pollution data are derived from the Shandong Provincial Public Data Open Network^1^ To accurately obtain the air quality data of the prefecture-level cities where the patients are located, this paper determines the admission dates and prefecture-level cities where the patients with T2DM are located from 2016 to 2021 and selects the observation data of the corresponding monitoring sites. Considering the time lag effect of patient onset, the specific admission date of each patient is backward 1 month, 3 months, and 6 months, and the average value of the corresponding daily air quality data is taken.

Thirdly, the inversion difference intensity data are obtained from the Modern-Era Retrospective Analysis for Research and Applications, Version 2 (MERRA-2) dataset, released by NASA's Global Modeling and Assimilation Office^2^. This dataset records the air temperature data of 42 atmospheric pressure layers (different altitudes have different atmospheric pressures) at a resolution of 0.5° × 0.625° in the unit of the month. This paper, quasi-change the raster data into urban panel data for analysis to meet the high requirements of data quality and precision (26). Specifically, this paper first calculate the average temperature of the atmosphere at 540 and 110 m, respectively and then calculates the difference between them. If the average temperature of the atmosphere at 540 m is higher than that of the atmosphere at 110 m, then the day is a temperature inversion (27).

Finally, since meteorological conditions can also affect the health of patients, it is necessary to control meteorological factors. First,we obtain the original natural meteorological data through the China Surface Climate Data Daily Value Dataset (V3.0)^3^, a national meteorological scientific data-sharing service platform. Then, we interpolate the original data into grid data using the Inverse Distance Weighted (IDW) method and calculate the average value in different regions. And last, we acquire multidimensional weather variables including annual average temperature (Temperature), average rainfall (Rainfall), Light hours (Light), and average wind speed (Wind) of the patient's location.

In addition, this paper also collects the economic and social development variables of the prefecture-level cities where the patients are located, including urbanization rate (Urbn), GDP per capita (PGDP), and the number of hospital beds per capita (Beds), which are derived from the China City Statistical Yearbook and the Statistical bulletin of prefecture-level cities.

### Model setting

The aim of this paper is to empirically examine the effects of air pollution on cardiovascular health in patients with T2DM. As mentioned above, the severity of the disease may vary depending on gender, age, weight, lifestyle, and key medical indicators. Climate change can also lead to imbalances between body functions and climate conditions. At the same time, the regional economic and social development conditions will also affect the physical quality of patients. Therefore, individual characteristics, climatic conditions, and regional development should be taken into account when assessing the severity of the disease. Therefore, the benchmark regression model of this paper is as follows:


$$\begin{array}{l} {Diseases}_{i,j,t}=\beta_{0}+\beta_{1}X_{i,j,t-1}+\gamma I_{i,j,t}+\mu C_{j,t}+\delta D_{j,t}+T_{t}+S_{i}+\varepsilon_{i,j,t} \end{array}$$

In Equation, the subscripts *i, j*, and *t* indicate the individual patient, the region, and the time, respectively; *Diseases* represent the severity of CVD in patients with T2DM; *X* indicates the degree of air pollution, the core explanatory variable of the model, which is represented by the concentration of PM2.5 and PM10 1 month before admission in this paper; *I, C* and *D* are control variables, which represent the individual characteristic variable, climate variable and regional economic and social characteristic variable of patients over time, respectively, and γ, μ, and δ are their coefficients, respectively. β_0_ is a characteristic variable of individual patients that does not change over time and region, and β_1_ is the core explanatory variable coefficient of this paper. In addition to this paper, we also controlled for region and time-fixed effects.

### Variable construction

#### Explained variable: Prevalence of CVD in T2DM patients

In this paper, we collect the disease diagnosis information of patients with T2DM from the Yi Du Cloud Medical Research Data Platform of the Provincial Hospital affiliated to Shandong First Medical University, including whether they had coronary heart disease, myocardial infarction, and hypertension, and add up the prevalence of these three CVDs. “None of the three diseases,” “one disease,” “two diseases” and “all three diseases” are assigned values 0 to 3, respectively. The higher the value, the more severe the CVD in patients with T2DM. Compared with a single measurement index, this method is more consistent with the reliability and validity of statistics.

#### Core explanatory variable: Air pollution

Air pollutants are composed of particulate matter and gaseous pollutants. According to the classification of aerodynamic equivalent diameter, particles with aerodynamic equivalent diameter ≤100 μm are collectively referred to as “total suspended particulate matter” (TSP), and particles with aerodynamic equivalent diameter less than or equal to 10 μm are collectively referred to as “inhalable particulate matter” (PM10). Particulate matter with an aerodynamic equivalent diameter of 2.5 μm or less is called fine particulate matter (PM2.5). Among them, PM2.5 and PM10 are the most intensively studied and extensively monitored. Gaseous pollutants include nitrogen oxides, such as nitrogen dioxide (NO_2_) and sulfur dioxide (SO_2_). Therefore, the concentration of PM2.5 and PM10 1 month before admission (PM2.5_1 and PM10_1) is selected as the air pollutant variables in the empirical analysis, and the SO_2_ concentration 1 month before admission (SO_2__1) is used to conduct the robustness test.

#### Other control variables

In this paper, we learn from previous studies on health influencing factors and introduce individual characteristics of patients as control variables, such as gender, age, body mass index (BMI), lifestyle habits (whether smoking or drinking), free triiodothyronine (FT3) level and blood urea nitrogen to creatinine ratio (BUN/Cr), to minimize estimation bias due to omitted variables. In addition, we control for city-level climate variables such as Temperature, Rainfall, Light, and Wind, and included city-level regional socio-economic characteristics such as Urbn, PGDP, and Beds. Table 1 shows the descriptive statistics of each variable in this study.

**Table 1:** Descriptive statistics (*n* = 1,446).

**Variable**	**Variable description**	**Mean**	**Standard**	**Min**	**Median**	**Max**
*Diseases*	Ordinal variable 0–3 (-)	0.8373	0.7460	0.0000	1.0000	3.0000
*PM*2.5_1	Continuous variable	0.5496	0.2496	0.1407	0.4917	1.6570
*PM*10_1	Continuous variable	1.0878	0.3927	0.2897	1.0680	2.3447
Individual level variables						
*Gender*	Male = 0, female = 1	0.4057	0.4912	0.0000	0.0000	1.0000
*Age*	Continuous variable (year)	57.8283	13.4488	10.5828	59.4099	93.0000
*BMI*	Continuous variable (kg/m^3^)	26.0277	6.8675	13.7000	25.6000	47.7000
*Smoking*	Yes = 1, No = 0	0.3947	0.4889	0.0000	0.0000	1.0000
*Drinking*	Yes = 1, No = 0	0.4323	0.4956	0.0000	0.0000	1.0000
*FT*_3_	Continuous variable (pmol/L)	4.3480	1.4857	1.4700	4.3000	30.2500
*BUN/Cr*	Continuous variable (-)	88.3680	29.5866	21.1300	84.4400	272.7300
Climate variables at the city level						
*Temprature*	Continuous variable (°C)	13.9190	0.6087	11.4635	13.8529	15.3437
*Rainfall*	Continuous variable (mm)	794.6238	100.8081	473.7313	828.1262	1100.0000
*Light*	Continuous variable (h)	2400.0000	144.1834	2000.0000	2400.0000	2900.0000
*Wind*	Continuous variable (m/s)	2.6993	0.3456	1.7827	2.7399	3.7847
Regional socio-economic characteristic variables						
*Urbn*	Continuous variable (-)	0.6699	0.0774	0.4736	0.7053	0.7717
*PGDP*	Continuous variable (RMB 10,000/person)	8.8783	2.7036	3.0028	9.8372	15.6797
*Beds*	Continuous variable (per thousand person)	6.5385	0.9754	3.7374	6.7614	8.2130

PM2.5_1 and PM10_1 represent PM2.5 and PM10 concentrations 1 month before admission, respectively; In the original data, the unit of PM2.5 and PM10 concentration is μg/m^3^. Considering the magnitude units of other variables, this paper divided the original concentration data by 100 in empirical regression.

**Figure 1:**
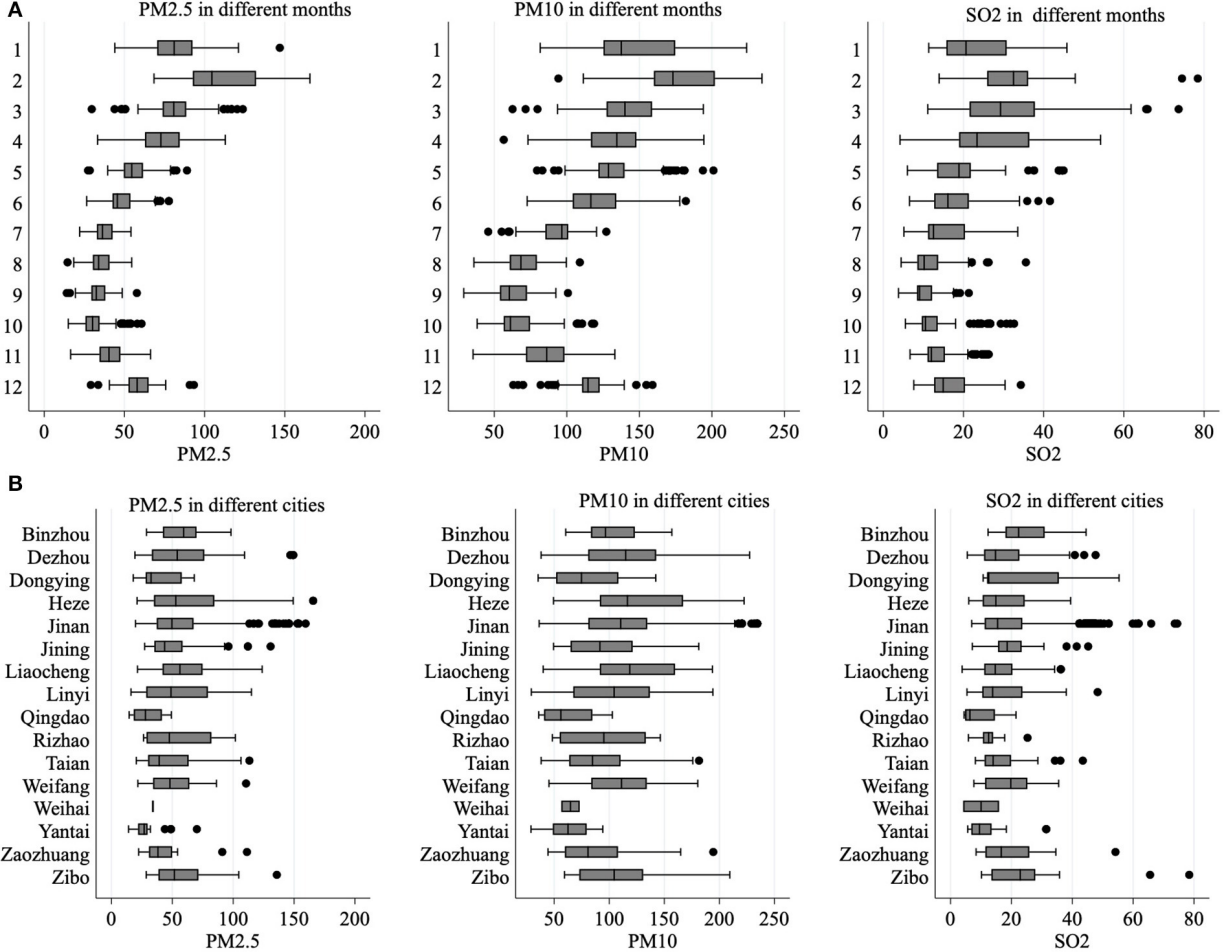
**(A,B)** Air pollution in different months and cities.

Figure 1 Further depicts the distribution of PM2.5, PM10, and SO_2_ concentrations in different months and in different cities. Figure 1A shows that the concentration of air pollutants varies greatly in different months, especially in winter. This is because Shandong Province is located to the north of Qinling Mountains and Huaihe River, and air pollutants will increase significantly during winter heating. Figure 1B shows that the air pollution in Jinan, Zaozhuang, and Dezhou is more serious, while the air quality in Weihai, Qingdao, and Yantai is better. In general, the concentration of air pollutants varies greatly in different months and cities, which meets the needs of empirical analysis.

## Results

### Benchmark regression

#### Impact of air quality on cardiovascular health of residents

This paper first estimates the overall impact of air pollution on the cardiovascular health of patients with T2DM according to the model set. Secondly, to reduce the endogenous estimation bias caused by the omitted variables, individual characteristics, regional climate, and economic and social characteristics are gradually controlled. The results are shown in Table 2. In column (1), this paper only adds air pollution variables and individual characteristic variables characterized by PM2.5 and estimates them based on a two-way fixed effect model. The results show that the coefficient of PM2.5_1 is significantly positive at the statistical level of 5%. The results in columns (2)–(3) show that the air pollution variable represented by PM2.5 is always significantly positive at the statistical level of 5% after successively adding climate variables such as Temperature, Rainfall, Light and Wind and regional socioeconomic characteristics variables such as Urbn, PGDP, and Beds. This indicates that the aggravation of air pollution will worsen the severity of CVD in patients with type 2 diabetes. From an economic perspective, for every 1 unit standard deviation increase in PM2.5_1, the severity of the patient's CVD will increase by a 5.58% standard deviation. Similar to the first three columns, control variables are also added in turn in columns (4)–(6). The difference is that PM10 is used to represent the air pollution variable. It can be seen from the results that the variable PM10_1 is always significantly positive at the statistical level of 5%, and hypothesis 1 is verified. For every 1 unit standard deviation increase in PM10_1, the severity of CVD of T2DM patients will increase by 5.70% standard deviation. The conclusion is still stable after changing different pollution indicators. Another interesting finding is that the absolute value of the estimated coefficient of PM2.5_1 is larger than the concentration coefficient of PM2.5_1. This is because PM2.5 is easier to enter the respiratory tract than PM10, which will cause harm to human cardiovascular function.

**Table 2:** Benchmark regression results.

**Variable**	**(1)**	**(2)**	**(3)**	**(4)**	**(5)**	**(6)**
*PM*2.5_1	0.1601**	0.1594**	0.1668**			
	(0.0807)	(0.0807)	(0.0807)			
	[0.0794]	[0.0794]	[0.0793]			
*PM*10_1				0.1033**	0.1048**	0.1083**
				(0.0512)	(0.0511)	(0.0511)
				[0.0513]	[0.0513]	[0.0512]
*Gender*	0.1236**	0.1222**	0.1233**	0.1228**	0.1212**	0.1222**
	(0.0542)	(0.0541)	(0.0542)	(0.0542)	(0.0541)	(0.0542)
*Age*	0.0193***	0.0195***	0.0196***	0.0194***	0.0196***	0.0197***
	(0.0015)	(0.0015)	(0.0015)	(0.0015)	(0.0015)	(0.0015)
*BMI*	0.0058	0.0057	0.0059	0.0060	0.0058	0.0060
	(0.0084)	(0.0083)	(0.0083)	(0.0084)	(0.0083)	(0.0083)
*Smoking*	−0.0226	−0.0187	−0.0176	−0.0232	−0.0193	−0.0183
	(0.0471)	(0.0471)	(0.0471)	(0.0472)	(0.0471)	(0.0472)
*Drinking*	0.0581	0.0573	0.0598	0.0592	0.0582	0.0607
	(0.0490)	(0.0490)	(0.0492)	(0.0490)	(0.0490)	(0.0492)
*FT*_3_	0.0059	0.0066	0.0067	0.0060	0.0067	0.0069
	(0.0165)	(0.0165)	(0.0164)	(0.0165)	(0.0166)	(0.0165)
*BUN/Cr*	−0.0020***	−0.0020***	−0.0021***	−0.0021***	−0.0021***	−0.0021***
	(0.0006)	(0.0006)	(0.0006)	(0.0006)	(0.0006)	(0.0006)
*Temperature*		−0.4868	−0.7673**		−0.5202	−0.7970**
		(0.3329)	(0.3555)		(0.3333)	(0.3567)
*Rainfall*		0.0043	0.0002		0.0044	0.0002
		(0.0053)	(0.0058)		(0.0053)	(0.0058)
*Light*		0.0004	0.0005		0.0003	0.0005
		(0.0004)	(0.0004)		(0.0004)	(0.0004)
*Wind*		−0.5329	−0.5108		−0.5208	−0.5037
		(0.3744)	(0.3915)		(0.3746)	(0.3914)
*Urbn*			−1.2292			−1.1347
			(2.5958)			(2.5939)
*PGDP*			−0.0609			−0.0593
			(0.0446)			(0.0446)
*Beds*			−0.2114**			−0.2113**
			(0.1043)			(0.1046)
*constant*	−0.4327	6.5522	13.0908**	−0.4643	6.9781	13.4034**
	(0.2830)	(5.0885)	(6.2712)	(0.2867)	(5.0874)	(6.2850)
*City FE*	*YES*	*YES*	*YES*	*YES*	*YES*	*YES*
*Year FE*	*YES*	*YES*	*YES*	*YES*	*YES*	*YES*
*N*	1,446	1,446	1,446	1,446	1,446	1,446
*Adj_R^2^*	0.1251	0.1252	0.1267	0.1251	0.1253	0.1267

Values in small brackets are heteroscedastic robust standard errors; values in middle brackets are spatially correlated robust standard errors. The *** and ** indicate that the regression results are significant at the 1% and 5% levels, respectively.

This paper first adopts the heteroscedasticity robust standard error. However, due to the impact of objective factors such as air pollution in various regions on the cardiovascular health of patients with T2DM in one region, it is bound to be affected by the neighboring regions, which presents the characteristics of mutual correlation. Therefore, the standard error of the regression coefficient may have a spatial correlation, which will affect the estimation result. Therefore, this paper further adopts the calculation method of spatial correlation robust standard error (28), and sets the critical value at 110 km to calculate the robust standard error. According to the results in Table 2, under the robust standard error of spatial correlation, the regression coefficients of PM2.5 concentration level and PM10 concentration level are still significantly positive at least at the level of 5%, and the standard deviation fluctuates slightly, so the results are relatively stable, which proves that air pollution does have a negative impact on the cardiovascular health of patients with T2DM.

#### Endogenous problem

Although the two-way fixed effect model and the addition of important control variables can partly solve the problem of missing variables that cannot be dealt with by the mixed ordinary least square (OLS) and random effect (RE) models, the problem of missing variables may still exist due to the complexity of the correlation between variables and the limitations of survey data. To solve this problem, this paper refers to the practice of Deschenes et al. (29) and adopts the inverse temperature difference days as the instrumental variable of air pollution to minimize the estimation error caused by endogenous problems and measurement errors. Throughout the existing research, many studies adopt this tool variable. For example, Arceo et al. (30) used the inversion difference days as an instrumental variable to empirically study the impact of air pollution on newborn infant mortality in Mexico; Deschenes et al. (29) used the instrumental variable identification strategy of temperature inversion difference and found that air pollution would lead to the deterioration of BMI and obesity-related indicators.

The selection of inversion days as an instrumental variable is mainly based on correlation and orthogonality (31). The first is a correlation, that is, inverse temperature difference must be related to PM2.5 concentration and PM10 concentration of air pollution. According to the principles of physics, the temperature in the troposphere will drop by 6.5°C every 1,000 m of altitude rise, and it will continue to climb with the airflow in the atmosphere. The suspended particles in the air will dissipate in mid-air with the thinning of the atmosphere. However, under certain conditions, the temperature in the troposphere will also rise with the increase in altitude, or the temperature change rate will be <6.5°C/1,000 m with the increase in ground altitude. This phenomenon is called “inversion.” This will cause the suspended particulate matter in the air to sink to the ground due to the obstruction of its longitudinal climb, thus increasing the concentration of PM2.5 and PM10. The suspended particles in the air will dissipate in mid-air with the thinning of the atmosphere. However, under certain conditions, the temperature in the troposphere will also rise with the increase in altitude, or the temperature change rate will be <6.5°C/1,000 m with the increase in ground altitude. This phenomenon is called “inversion.” This will cause the suspended particulate matter in the air to sink to the ground due to the obstruction of its longitudinal climb, thus increasing the concentration of PM2.5 and PM10. Previous studies have shown that the “inversion” phenomenon in the troposphere structure is highly correlated with the concentration of PM2.5 and PM10 in air pollution, and this conclusion has been verified by the analytical charts of meteorological observation fields around the world (32). The second is orthogonality, that is, the intensity of inverse temperature difference will affect the severity of CVD, but the latter will not affect the former in turn. This is because “temperature inversion” is a natural weather phenomenon with exogenous impact and randomness, the individual's level of CVD of individuals cannot play a role in it. Although China's economic growth rate and air pollution intensity rose sharply after 2001, the inverse temperature difference intensity did not change significantly, which further confirmed the orthogonality between the two (32). In addition, in order to further avoid the interference of natural environmental factors and economic and social development factors on the regression of instrumental variables in the first stage, the control variables in the benchmark regression are added in this paper.

**Figure 2:**
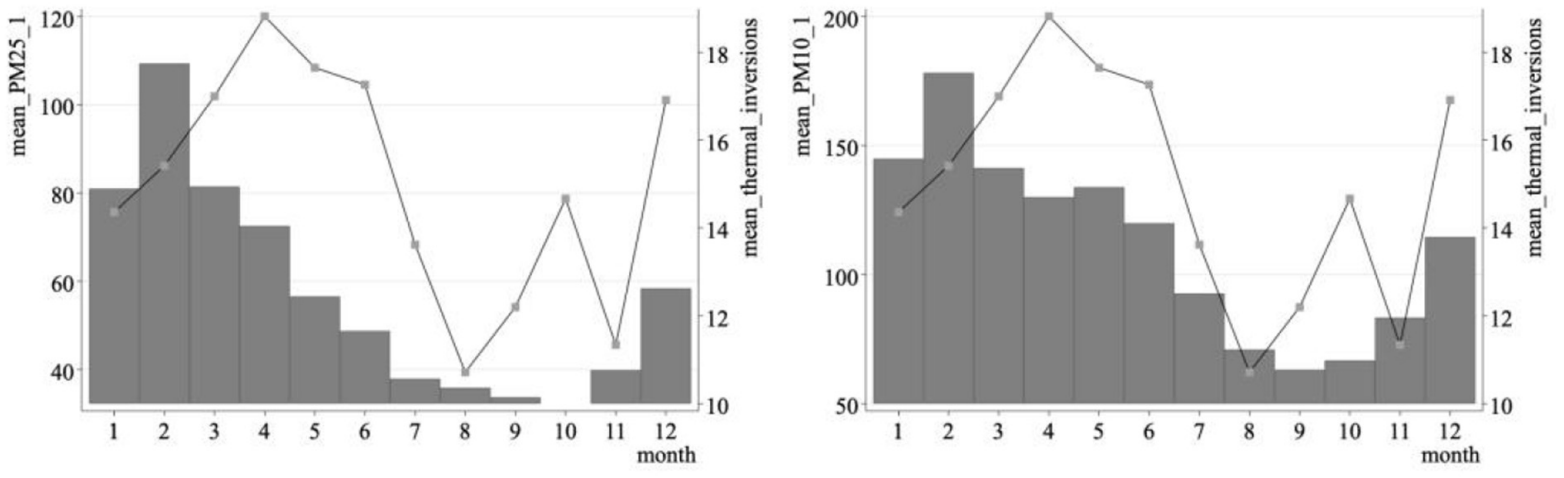
The relationship between the number of inversion days per month and air pollution.

First of all, this paper studies the relationship between the occurrence of temperature inversion and two air pollutants. In Figure 2, the average pollution level is plotted according to the monthly average inversion days. As shown in the figure, there is a strong positive correlation between inversion days and PM2.5 and PM10 in air pollutants. To further prove the existence of this relationship, in the first stage of the instrumental variable method, this paper takes two air pollutants as explained variables.

From columns (1)–(2) of Table 3, the regression coefficient of the influence of the days when the inversion phenomenon occurs on air pollution has passed the significance test at the level of 1%, which indicates that the inversion phenomenon will indeed worsen the air pollutant index. And all passed the identification deficiency and weak instrumental variable test. From columns (3) to (4) of Table 3, for each increase of 1 unit standard deviation of PM2.5 and PM10 concentrations in air pollutants, the severity of CVD of patients will increase by 24.96 and 19.64%, respectively. The coefficients of all core explanatory variables are larger than those estimated by OLS in Table 2, indicating that endogenous problems will lead to underestimation to a certain extent.

**Table 3:** Effects of air pollution on cardiovascular health [Instrumental variable (IV) estimation model].

**Variable**	***PM*** **2.5_1**	***PM*** **10_1**	***Diseases***	***Diseases***
	**(1)**	**(2)**	**(3)**	**(4)**
*Thermal_inversions*	0.0088***	0.0176***		
	(0.0012)	(0.0018)		
*PM*2.5_1			0.7460*	
			(0.4126)	
*PM*10_1				0.3731*
				(0.2051)
*Gender*	0.0029	0.0087	0.1173**	0.1162**
	(0.0180)	(0.0275)	(0.0560)	(0.0553)
*Age*	−0.0006	−0.0016**	0.0199***	0.0201***
	(0.0005)	(0.0007)	(0.0015)	(0.0015)
*BMI*	0.0005	−0.0002	0.0053	0.0058
	(0.0009)	(0.0014)	(0.0073)	(0.0077)
*Smoking*	−0.0094	−0.0089	−0.0099	−0.0136
	(0.0164)	(0.0251)	(0.0483)	(0.0477)
*Drinking*	0.0074	−0.0010	0.0608	0.0667
	(0.0167)	(0.0255)	(0.0503)	(0.0496)
*FT*_3_	−0.0026	−0.0052	0.0076	0.0076
	(0.0043)	(0.0066)	(0.0166)	(0.0169)
*BUN/Cr*	−0.0002	−0.0001	−0.0019***	−0.0020***
	(0.0002)	(0.0003)	(0.0006)	(0.0006)
*Temperature*	−0.0612	0.1864	−0.6631*	−0.7782**
	(0.1237)	(0.1891)	(0.3679)	(0.3694)
*Rainfall*	0.0007	−0.0002	−0.0012	−0.0006
	(0.0020)	(0.0030)	(0.0061)	(0.0059)
*Light*	−0.0002	−0.0001	0.0006	0.0006
	(0.0001)	(0.0002)	(0.0004)	(0.0004)
*Wind*	0.0206	−0.0226	−0.4408	−0.4171
	(0.1301)	(0.1989)	(0.4101)	(0.4000)
*Urbn*	0.6767	0.2069	−1.8908	−1.4624
	(0.8460)	(1.2935)	(2.6759)	(2.6128)
*PGDP*	−0.0049	−0.0296	−0.0680	−0.0607
	(0.0160)	(0.0245)	(0.0468)	(0.0461)
*Beds*	0.0330	0.0431	−0.2324**	−0.2238**
	(0.0343)	(0.0524)	(0.1086)	(0.1074)
*constant*	0.9643	−1.4981		
	(2.1114)	(3.2281)		
*Kleibergen-Paap rk LM*			63.3930	97.9980
			(0.0000)	(0.0000)
*Kleibergen-Paap rk Wald F*			73.3980	125.0250
			(16.3800)	(16.3800)
*City FE*	*YES*	*YES*	*YES*	*YES*
*Year FE*	*YES*	*YES*	*YES*	*YES*
*N*	1,446	1,446	1,446	1,446
*Adj_R^2^*	0.1722	0.2187	0.0853	0.1020

The parenthesis content of Kleibergen-Paaprk LM statistic is the *p*-value of the insufficient identification test, the parenthesis content of Kleibergen-Paaprk Wald F statistic is the 10% critical value of the weak identification test, and the rest of the parenthesis content is the heteroskedvariance robust standard error. The ***, ** and * indicate that the regression results are significant at the 1%, 5% and 10% levels, respectively.

#### Robust test

##### Robust test for adjusted variables

First, this paper replaces the core explanatory variables. A 9-year epidemiological cohort study of hypertension covering about 70,000 people in 31 cities in China found that an increase of 10 μg/m^3^ in SO_2_ and TSP was associated with an increase of 3.2 and 0.9% in cardiovascular mortality, respectively (33). Moreover, in addition to its own toxicity, SO_2_, a gaseous pollutant, can also contribute to the generation of PM2.5 through complex photochemical reactions and form secondary pollution. Therefore, it is reasonable to select SO_2__1 to measure the degree of air pollution and use it to replace PM2.5 and PM10, which can be used as the basis for the robustness test.

Second, this paper extends the time length of core explanatory variables. In the benchmark regression, PM2.5_1 and PM10_1 were respectively used to characterize the degree of air pollution. However, considering that most CVDs are due to long-term exposure to air pollutants, the test period for air pollution before the patient's admission is extended in the robustness test, and the test period for core explanatory variables is extended to 3 months.

The robustness test results of the adjusted variables are shown in columns (1) to (3) of Table 4. Table 4 lists the regression results of replacing the core explanatory variables, prolonging the investigation time of the core explanatory variables, and replacing the explained variables. In columns (1) to (3) of Table 4, the regression coefficients of the core explanatory variables have changed significantly at the sign and significance levels, which further confirms the results and explanations in the benchmark regression. Specifically, from the regression coefficient of air pollution degree in column (1), the air pollution represented by SO_2_ worsens the severity of CVD in patients with T2DM, indicating that the change in direction of gaseous pollutants and particulate pollutants and their impact on CVD are similar. The regression results in columns (2)–(3) that when the investigation period of air pollution is extended, the significance of the regression coefficient does not change, but the absolute value of the coefficient decreases, indicating that the influence effect will gradually decrease with time.

**Table 4:** Robustness test of substitution variables and transformation regression method.

**Variable**	***Diseases***	***Diseases***	***Diseases***	***OProbit***		***OLogit***	
	**(1)**	**(2)**	**(3)**	**(4)**	**(5)**	**(6)**	**(7)**
*SO*_2__1	0.0055**						
	(0.0022)						
*PM*2.5*_*3		0.0027***					
		(0.0009)					
*PM*10*_*3			0.0014**				
			(0.0006)				
*PM*2.5*_*1				0.2704**		0.4489*	
				(0.1299)		(0.2327)	
*PM*10_1					0.1783**		0.3122**
					(0.0836)		(0.1468)
*Gender*	0.1208**	0.1218**	0.1223**	0.1987**	0.1968**	0.3237**	0.3201**
	(0.0542)	(0.0542)	(0.0541)	(0.0887)	(0.0887)	(0.1565)	(0.1564)
*Age*	0.0195***	0.0196***	0.0196***	0.0327***	0.0328***	0.0586***	0.0589***
	(0.0015)	(0.0015)	(0.0015)	(0.0026)	(0.0026)	(0.0061)	(0.0061)
*BMI*	0.0059	0.0061	0.0062	0.0094	0.0096	0.0598	0.0605
	(0.0083)	(0.0083)	(0.0084)	(0.0124)	(0.0125)	(0.0406)	(0.0405)
*Smoking*	−0.0190	−0.0194	−0.0182	−0.0315	−0.0327	−0.0763	−0.0763
	(0.0471)	(0.0471)	(0.0472)	(0.0776)	(0.0777)	(0.1346)	(0.1348)
*Drinking*	0.0581	0.0616	0.0611	0.1164	0.1183	0.1466	0.1474
	(0.0491)	(0.0492)	(0.0493)	(0.0817)	(0.0818)	(0.1486)	(0.1486)
*FT*_3_	0.0063	0.0062	0.0061	0.0111	0.0114	0.0021	0.0021
	(0.0167)	(0.0165)	(0.0165)	(0.0289)	(0.0290)	(0.0469)	(0.0471)
*BUN/Cr*	−0.0021***	−0.0021***	−0.0021***	−0.0034***	−0.0034***	−0.0053***	−0.0054***
	(0.0006)	(0.0006)	(0.0006)	(0.0010)	(0.0010)	(0.0018)	(0.0018)
*Temperature*	−0.7491**	−0.7725**	−0.8071**	−1.2442**	−1.2908**	−1.9107*	−2.0048*
	(0.3555)	(0.3558)	(0.3565)	(0.5910)	(0.5927)	(1.0271)	(1.0331)
*Rainfall*	−0.0003	0.0005	0.0006	−0.0005	−0.0003	−0.0041	−0.0040
	(0.0058)	(0.0058)	(0.0058)	(0.0096)	(0.0096)	(0.0163)	(0.0163)
*Light*	0.0005	0.0005	0.0005	0.0010	0.0009	0.0014	0.0013
	(0.0004)	(0.0004)	(0.0004)	(0.0007)	(0.0007)	(0.0013)	(0.0013)
*Wind*	−0.4858	−0.5330	−0.5117	−0.8666	−0.8533	−1.2617	−1.2309
	(0.3931)	(0.3927)	(0.3918)	(0.6384)	(0.6382)	(1.1193)	(1.1190)
*Urbn*	−1.0490	−1.4515	−1.2914	−1.4655	−1.3159	−1.2328	−0.9006
	(2.5969)	(2.6076)	(2.6019)	(4.4013)	(4.3998)	(7.4775)	(7.4730)
*PGDP*	−0.0557	−0.0571	−0.0562	−0.0972	−0.0947	−0.1567	−0.1527
	(0.0455)	(0.0447)	(0.0447)	(0.0751)	(0.0752)	(0.1305)	(0.1307)
*Beds*	−0.2157**	−0.2118**	−0.2092**	−0.3551**	−0.3552**	−0.6775**	−0.6830**
	(0.1050)	(0.1051)	(0.1049)	(0.1726)	(0.1731)	(0.3051)	(0.3063)
*constant*	12.7562**	13.2245**	13.5515**				
	(6.2599)	(6.2793)	(6.2798)				
*City FE*	*YES*	*YES*	*YES*	*YES*	*YES*	*YES*	*YES*
*Year FE*	*YES*	*YES*	*YES*	*YES*	*YES*	*YES*	*YES*
*N*	1,446	1,446	1,446	1,446	1,446	1,446	1,446
*Adj*_*R*^2^/*Pseudo*_*R*^2^	0.1283	0.1297	0.1272	0.0750	0.0751	0.0792	0.0794

PM2.5_3, PM10_3 and SO_2__3 represent PM2.5, PM10 and SO_2_ concentrations 3 months before admission, respectively; Values in parentheses are heteroscedastic robust standard errors; The ***, ** and * indicate that the regression results are significant at the 1%, 5% and 10% levels, respectively.

##### Robustness test of transformation regression method

In the benchmark regression in this paper, the explained variable is the CVDs of patients with T2DM. The measurement method is to add the prevalence of coronary heart disease, myocardial infarction, and hypertension to obtain a discrete integer variable, which has internal ranking characteristics. Therefore, it is necessary to use maximum likelihood estimation to estimate the ranking model. Assuming that the random disturbance term obeys the normal distribution, OProbit model is selected for estimation; It is more reasonable to use the ordered logit (OLogit) model if the random disturbance term obeys the logical distribution. In this paper, the OProbit and OLogit models are successively used to estimate the benchmark regression in the robustness test under the two scenarios where PM2.5 and PM10 concentrations are used to measure the air pollution status. From the regression results in columns (4) to (7) of Table 4 whether the Oprobit model or the OLogit model is used, the air pollution variables pass the significance test and the coefficient value is not much different from the benchmark regression, which further confirms the hypothesis that air pollution will worsen the CVD.

### Analysis of heterogeneity

Above, this paper estimates the average effect of air pollution on the cardiovascular health of patients with T2DM. It is assumed that all individuals and cities are affected by the same impact. However, the air pollution level among cities is not evenly distributed, and there is heterogeneity among different cities, which may lead to differences in the impact of air pollution on the cardiovascular health of patients with T2DM. Therefore, this paper conducts a sub-sample test on the benchmark model from the perspectives of gender, age, environmental regulation, and medical and health conditions. Considering that the estimator obeys a certain distribution, and its confidence intervals overlap, this paper tests the difference of coefficients between groups. The results are shown in Table 5.

**Table 5:** Heterogeneity analysis of gender.

**Variable**	***PM*** **2.5_1**		***PM*** **10_1**	
	**Male**	**Female**	**Male**	**Female**
	**(1)**	**(2)**	**(3)**	**(4)**
*PM*2.5*_*1	0.2206**	0.0419		
	(0.1026)	(0.1326)		
*PM*10_1			0.1373**	0.0535
			(0.0654)	(0.0841)
*Age*	0.0177***	0.0237***	0.0178***	0.0237***
	(0.0018)	(0.0022)	(0.0018)	(0.0022)
*BMI*	0.0015	0.0338***	0.0017	0.0338***
	(0.0060)	(0.0075)	(0.0060)	(0.0075)
*Smoking*	−0.0270	0.0019	−0.0281	0.0001
	(0.0502)	(0.1445)	(0.0503)	(0.1446)
*Drinking*	0.0591	0.0380	0.0607	0.0402
	(0.0524)	(0.1524)	(0.0524)	(0.1520)
*FT*_3_	0.0059	0.0098	0.0065	0.0099
	(0.0174)	(0.0238)	(0.0175)	(0.0239)
*BUN/Cr*	−0.0018**	−0.0021**	−0.0019**	−0.0021**
	(0.0009)	(0.0009)	(0.0009)	(0.0009)
*Temperature*	−0.9563**	−0.6278	−0.9926**	−0.6360
	(0.4081)	(0.8524)	(0.4113)	(0.8513)
*Rainfall*	0.0002	−0.0112	0.0002	−0.0112
	(0.0069)	(0.0105)	(0.0069)	(0.0106)
*Light*	0.0007	−0.0006	0.0006	−0.0006
	(0.0005)	(0.0009)	(0.0005)	(0.0009)
*Wind*	−0.2712	−0.6526	−0.2734	−0.6531
	(0.4813)	(0.6908)	(0.4802)	(0.6892)
*Urbn*	−0.4124	−7.7849*	−0.2654	−7.7822*
	(3.2192)	(4.6603)	(3.2109)	(4.6638)
*PGDP*	−0.0701	−0.0221	−0.0690	−0.0220
	(0.0555)	(0.0717)	(0.0556)	(0.0722)
*Beds*	−0.2142*	−0.3305*	−0.2143	−0.3314*
	(0.1295)	(0.1693)	(0.1303)	(0.1693)
*constant*	14.4091**	19.3941	14.8211**	19.4763
	(7.0019)	(15.8317)	(7.0342)	(15.8175)
*City FE*	*YES*	*YES*	*YES*	*YES*
*Year FE*	*YES*	*YES*	*YES*	*YES*
*Experience P-value*	0.0020	0.0260
*N*	864	582	864	582
*Adj*_*R*^2^	0.0933	0.1641	0.0927	0.1646

Values in parentheses are heteroscedastic robust standard errors. The ***, ** and * indicate that the regression results are significant at the 1%, 5% and 10% levels, respectively.

#### Individual level: Heterogeneity analysis based on individual sex

To investigate whether there is a gender difference in the cardiovascular health of T2DM patients caused by air pollution, this paper conducts group regressions by gender based on benchmark regression. The results show that the regression coefficients of air pollution are all positive, but the effect is not significant in the female group, indicating that air pollution has a greater impact on the cardiovascular health of male patients with T2DM. It may be that compared with women, men are more likely to engage in outdoor labor and are more likely to be exposed to air pollution for a long time, so they are more sensitive to air pollution than women.

#### Individual level: Heterogeneity analysis based on individual age

To verify whether there is an age difference in the effect of air pollution on the cardiovascular health of patients with T2DM, this paper conducts group regression according to age on the basis of benchmark regression. The population aged 60 and above are classified as the elderly population, and the population under 60 is classified as the young and middle-aged population. The results are shown in Table 6. The results show that the regression coefficients of air pollution are all positive, but the effect is not significant in the young and middle-aged samples, indicating that air pollution has a greater impact on the cardiovascular health of elderly patients with T2DM aged 60 years and above. It may be due to the better physical fitness of young and middle-aged groups, so air pollution has less impact on them. On the contrary, the elderly will experience a certain degree of degradation in various bodily functions and are more susceptible to the effects of air pollution, which can lead to the deterioration of cardiovascular health. In response to the current situation of accelerating aging and worrying residents' health in China, all regions should strengthen residents' attention to the health of the elderly population and reduce the negative effects of air pollution on the health of vulnerable elderly people.

**Table 6:** Heterogeneity analysis of age.

**Variable**	**PM2.5_1**		***PM*** **10_1**	
	**Young and middle-aged**	**The elderly**	**Young and middle-aged**	**The elderly**
	**(1)**	**(2)**	**(3)**	**(4)**
*PM*2.5*_*1	0.0544	0.3175**		
	(0.0976)	(0.1320)		
*PM*10_1			0.0642	0.1738**
			(0.0612)	(0.0836)
*Gender*	0.1384**	0.1016	0.1400**	0.0977
	(0.0654)	(0.0859)	(0.0654)	(0.0861)
*Age*	0.0194***	0.0197***	0.0195***	0.0198***
	(0.0024)	(0.0042)	(0.0024)	(0.0042)
*BMI*	0.0301***	0.0004	0.0300***	0.0005
	(0.0054)	(0.0058)	(0.0054)	(0.0058)
*Smoking*	−0.0207	−0.0192	−0.0203	−0.0162
	(0.0570)	(0.0796)	(0.0570)	(0.0797)
*Drinking*	0.0955	−0.0045	0.0955	−0.0075
	(0.0586)	(0.0839)	(0.0587)	(0.0841)
*FT*_3_	−0.0221**	0.0463	−0.0220**	0.0474
	(0.0087)	(0.0296)	(0.0086)	(0.0294)
*BUN/Cr*	−0.0018**	−0.0020**	−0.0018**	−0.0021**
	(0.0008)	(0.0009)	(0.0008)	(0.0009)
*Temperature*	−0.5814	−0.7141	−0.6076	−0.7671
	(0.4290)	(0.6689)	(0.4312)	(0.6769)
*Rainfall*	0.0017	−0.0037	0.0017	−0.0025
	(0.0078)	(0.0091)	(0.0077)	(0.0091)
*Light*	0.0008	−0.0001	0.0007	−0.0000
	(0.0005)	(0.0006)	(0.0005)	(0.0006)
*Wind*	−0.1548	−1.2165*	−0.1435	−1.1639*
	(0.5409)	(0.6345)	(0.5428)	(0.6310)
*Urbn*	1.2232	−6.7049	1.2594	−6.3999
	(3.0232)	(4.5779)	(3.0208)	(4.6068)
*PGDP*	−0.0924	0.0115	−0.0912	0.0069
	(0.0568)	(0.0780)	(0.0568)	(0.0782)
*Beds*	−0.1021	−0.2613	−0.1063	−0.2497
	(0.1235)	(0.1737)	(0.1241)	(0.1744)
*constant*	6.2627	19.3059*	6.5838	19.4784*
	(7.9174)	(10.9984)	(7.9423)	(11.1190)
*City FE*	*YES*	*YES*	*YES*	*YES*
*Year FE*	*YES*	*YES*	*YES*	*YES*
*Experience P-value*	0.0000		0.0320	
*N*	716	730	716	730
*Adj*_*R*^2^	0.0992	0.0368	0.1002	0.0345

Values in parentheses are heteroscedastic robust standard errors. The ***, ** and * indicate that the regression results are significant at the 1%, 5% and 10% levels, respectively.

#### External environment: Heterogeneity analysis based on environmental regulation

To analyze the negative effects of air pollution on the cardiovascular health of patients with T2DM, it is necessary to find a breakthrough to reduce the negative effects of air pollution in combination with the actual situation of China at this stage, to provide more enlightenment for policy formulation. Therefore, this paper considers the perspective of the intensity of government environmental regulation, divides the samples into a low environmental regulation group and high environmental regulation group according to the intensity of urban environmental regulation, and conducts regression in sub-samples. The intensity of environmental regulation is measured by the proportion of local government energy conservation and environmental protection expenditure in GDP. The results are shown in Table 7.

**Table 7:** Heterogeneity analysis of environmental regulation.

**Variable**	***PM*** **2.5_1**		***PM*** **10_1**	
	**Low environmental regulation group**	**High environmental regulation group**	**Low environmental regulation group**	**High environmental regulation group**
	**(1)**	**(2)**	**(3)**	**(4)**
*PM*2.5*_*1	0.3799***	0.0796		
	(0.1356)	(0.1015)		
*PM*10_1			0.1949**	0.0582
			(0.0796)	(0.0678)
*Gender*	0.0802	0.1278*	0.0759	0.1272*
	(0.0753)	(0.0766)	(0.0757)	(0.0765)
*Age*	0.0221***	0.0189***	0.0224***	0.0190***
	(0.0020)	(0.0019)	(0.0020)	(0.0019)
*BMI*	0.0285***	0.0025	0.0296***	0.0025
	(0.0068)	(0.0066)	(0.0068)	(0.0066)
*Smoking*	−0.1150*	0.0062	−0.1117	0.0051
	(0.0679)	(0.0641)	(0.0682)	(0.0641)
*Drinking*	0.2011***	−0.0458	0.1975***	−0.0448
	(0.0700)	(0.0677)	(0.0704)	(0.0677)
*FT*_3_	0.0051	0.0110	0.0051	0.0110
	(0.0172)	(0.0238)	(0.0173)	(0.0239)
*BUN/Cr*	−0.0016**	−0.0024***	−0.0017**	−0.0024***
	(0.0008)	(0.0009)	(0.0008)	(0.0009)
*Temperature*	−1.7701**	−1.1059	−1.8468**	−1.1005
	(0.8944)	(1.3887)	(0.8916)	(1.3876)
*Rainfall*	−0.0011	−0.0115	0.0006	−0.0115
	(0.0130)	(0.0136)	(0.0132)	(0.0137)
*Light*	0.0006	0.0014	0.0007	0.0014
	(0.0009)	(0.0024)	(0.0009)	(0.0024)
*Wind*	2.7920	1.2570	2.5656	1.2424
	(1.8339)	(0.8540)	(1.8432)	(0.8537)
*Urbn*	3.6516	−4.5053	3.1506	−4.4762
	(7.9851)	(7.0608)	(7.9717)	(7.0527)
*PGDP*	0.0072	0.0274	−0.0013	0.0274
	(0.1087)	(0.1275)	(0.1094)	(0.1277)
*Beds*	−0.3609**	0.2421	−0.3560**	0.2312
	(0.1571)	(0.4825)	(0.1574)	(0.4844)
*constant*	13.6960	10.8143	15.3991	10.7702
	(16.1330)	(20.6708)	(16.1204)	(20.6311)
*City FE*	*YES*	*YES*	*YES*	*YES*
*Year FE*	*YES*	*YES*	*YES*	*YES*
*Experience P-value*	0.0000		0.0100	
*N*	615	831	615	831
*Adj*_*R*^2^	0.1662	0.1131	0.1638	0.1132

Values in parentheses are heteroscedastic robust standard errors. The ***, ** and * indicate that the regression results are significant at the 1%, 5% and 10% levels, respectively.

From the regression results in Table 7, the influence of air pollution on the severity of CVD in patients with T2DM is all positive, but it is more significant in the sample group with low environmental regulation. This paper further observes the coefficient of air particulate pollution in each sub-sample. The results show that from the perspective of PM2.5 concentration, for each unit standard deviation increase, the severity of CVD in the high and low environmental regulation groups will increase by 12.71 and 2.67% standard deviation, respectively. In addition, from the perspective of PM10 concentration, for every standard deviation increase of 1 unit, the severity of CVD in the high and low environmental regulation groups will increase by 10.07 and 3.06% standard deviations, respectively, which indicates that air pollution affects the health of the low environmental regulation groups. The negative effect is greater than that of high environmental regulation groups. It also further proves that the improvement of environmental regulation intensity helps to reduce the negative effect of air pollution on the cardiovascular health of patients with T2DM and proves the necessity of environmental governance. Facing the current situation of air quality deterioration in China, we should pay attention to enhancing the intensity of environmental regulation to curb the negative effects of air pollution.

#### External environment: Heterogeneity analysis based on medical and health conditions

The average effect of air pollution on cardiovascular health of T2DM patients has been discussed in this paper. However, the current medical and health conditions in China are unevenly distributed, and the ability to avoid environmental negative externalities is obviously different in different medical and health level areas. It is necessary to further study whether there are significant differences in the health effects of air pollution on different groups with different ability to avoid negative environmental externalities. Therefore, according to the level of urban medical and health care, this paper divides the samples into two groups: poor medical conditions and excellent medical conditions and conducts regression analysis by groups. The level of medical and health conditions is measured by the proportion of local government health expenditure in GDP. The results are shown in Table 8.

**Table 8:** Heterogeneity analysis of medical and health conditions.

**Variable**	***PM*** **2.5_1**		***PM*** **10_1**	
	**Poor medical conditions group**	**Excellent medical conditions group**	**Poor medical conditions group**	**Excellent medical conditions group**
	**(1)**	**(2)**	**(3)**	**(4)**
*PM*2.5*_*1	0.2746**	0.1082		
	(0.1293)	(0.1043)		
*PM*10_1			0.1978***	0.0399
			(0.0748)	(0.0700)
*Gender*	0.1102	0.1140	0.1082	0.1134
	(0.0751)	(0.0795)	(0.0751)	(0.0793)
*Age*	0.0217***	0.0192***	0.0219***	0.0192***
	(0.0019)	(0.0020)	(0.0018)	(0.0020)
*BMI*	0.0314***	0.0012	0.0317***	0.0013
	(0.0063)	(0.0060)	(0.0063)	(0.0060)
*Smoking*	−0.0193	−0.0251	−0.0184	−0.0274
	(0.0684)	(0.0661)	(0.0683)	(0.0661)
*Drinking*	0.0286	0.0728	0.0252	0.0754
	(0.0680)	(0.0727)	(0.0680)	(0.0728)
*FT*_3_	0.0106	0.0084	0.0111	0.0084
	(0.0171)	(0.0239)	(0.0171)	(0.0240)
*BUN/Cr*	−0.0014*	−0.0025***	−0.0014*	−0.0025***
	(0.0008)	(0.0009)	(0.0008)	(0.0009)
*Temperature*	0.5241	−1.0106*	0.4636	−1.0259*
	(2.1018)	(0.5600)	(2.1573)	(0.5621)
*Rainfall*	−0.0017	0.0027	−0.0010	0.0028
	(0.0186)	(0.0091)	(0.0190)	(0.0091)
*Light*	0.0009	0.0007	0.0009	0.0007
	(0.0012)	(0.0005)	(0.0012)	(0.0005)
*Wind*	2.1320	0.8891	2.1158	0.8230
	(1.5920)	(1.3760)	(1.6338)	(1.3783)
*Urbn*	13.1026	−0.1984	13.1735	−0.2396
	(21.1146)	(3.2903)	(21.5422)	(3.2889)
*PGDP*	0.0808	0.0832	0.0790	0.0820
	(0.1774)	(0.0945)	(0.1814)	(0.0946)
*Beds*	−0.1114	−0.1701	−0.1321	−0.1699
	(0.3325)	(0.2029)	(0.3383)	(0.2033)
*constant*	−26.1183	10.2580	−25.3603	10.7526
	(25.9033)	(8.9120)	(26.2637)	(8.9178)
*City FE*	*YES*	*YES*	*YES*	*YES*
*Year FE*	*YES*	*YES*	*YES*	*YES*
*Experience P-value*	0.0280		0.0000	
*N*	702	743	702	743
*Adj*_*R*^2^	0.1591	0.1078	0.1619	0.1069

Values in parentheses are heteroscedastic robust standard errors. The ***, ** and * indicate that the regression results are significant at the 1%, 5% and 10% levels, respectively.

The regression results in Table 8 show that air pollution, whether expressed in terms of PM2.5 or PM10 concentration, has a negative impact on the cardiovascular health of the sample groups with different air pollution avoidance abilities, but the impact is more significant in the sample group with poor medical and health conditions. This paper further compares the pollution coefficients of groups with poor and good medical and health conditions. It can be found that, from the perspective of PM2.5 concentration, for everyone standard deviation increase of PM2.5 concentration, the severity of CVD in poor and good medical and health conditions will increase by 9.02 and 3.62% standard deviations, respectively. From the point of view of PM10 concentration, the severity of CVD in high and low environmental regulation groups will increase by 10.41 and 2.10% standard deviation, respectively, when the concentration of PM10 increases by 1 standard deviation. This indicates that the negative effect of air pollution on the health of people in areas with poor medical and health conditions is greater than that of people in areas with good medical and health conditions.

## Conclusions and implications

This paper analyzes the impact of air pollution on the cardiovascular health of patients with T2DM and its group differences and makes an empirical test by matching the daily air quality monitoring data of 16 prefecture-level cities of Shandong Provincial Public Data Open Network with the micro-data of the Provincial Hospital affiliated to Shandong First Medical University from 2016 to 2021. The results show that: (1) Air pollution will worsen the CVD of T2DM patients, that is, cardiovascular health declines in people with T2DM as regional air pollution levels rise. Spatial correlation robust standard error is used to solve the spatial spillover effect of air pollution and the inversion phenomenon is used as an instrumental variable to effectively solve the endogenous problem of air pollution. The results still significantly prove the robustness of the research conclusion. (2) From the perspective of individual characteristics, the impact of air pollution on different gender and age groups is different. Compared with women, men are more susceptible to the negative health effects of air pollution, the elderly bear greater health losses from air pollution than the young and middle-aged due to physical degradation. (3) From the external environment, areas with high environmental regulation intensity will significantly reduce the negative impact of air pollution on cardiovascular health, which further illustrates the necessity of environmental governance; The negative effects of air pollution on health can be alleviated in areas with good public health conditions, which indicates that the improvement of public health service conditions can provide a certain barrier for the cardiovascular health of patients with T2DM.

CVD is a leading cause of global mortality and morbidity. Quantitative assessment of the impact of environmental quality on the cardiovascular health of patients with T2DM not only helps to understand the social welfare cost caused by air pollution more comprehensively but also has important implications for environmental governance and the health effect of public health investment. Therefore, the conclusion contains rich policy implications: (1) Policymakers should optimize the economic development mode, take people-oriented as the development purpose, treat the relationship between economy and environment from the perspective of development, no longer follow the old road of “pollution first, treatment later,” and must fully consider the impact of economic development on the environment and residents' health. (2) The government should improve the CVD prevention system, increase the publicity of CVD prevention, inform the public, especially men and middle-aged and elderly groups, of the hazards of air pollution, and pay attention to preventing air pollution. (3) Environmental regulation and public health services can be used as a breakthrough to curb the negative impact of air pollution on health. Increasing the intensity of environmental regulation and public health expenditure will effectively reduce the negative impact of air pollution on the cardiovascular health of patients with T2DM and achieve economic-environmental coordinated development.

There are some potential limitations in this paper. First, the health data are collected from a single health care facility, which may limit the generality of the findings. Although Shandong Provincial Hospital Affiliated to Shandong First Medical University is one of the largest tertiary hospitals in Shandong Province, and its endocrinology is national key clinical specialty, the study sample may still be underrepresented, and it is necessary to further expand the sample study in the future to provide evidence. Second, adolescents or children are also vulnerable groups to air pollution, but this was not analyzed due to data availability. Analysis of the specific impact of air pollution for adolescents, children and middle-aged groups should be the direction of further exploration in the future. In addition, air pollution not only affects the cardiovascular health of diabetic patients, but also has a very important effect on individual mental health and economic behavior. The research on these issues is beyond the scope of this paper and needs to be further studied.

## Data availability statement

The raw data supporting the conclusions of this article will be made available by the authors, without undue reservation.

## Ethics statement

The studies involving human participants were reviewed and approved by Biomedical Research Ethic Committee of Shandong Provincial Hospital (Approval No. SWYX2022472). Written informed consent from the participants' legal guardian/next of kin was not required to participate in this study in accordance with the national legislation and the institutional requirements.

## Author contributions

JZ: wringing–original drift, writing–review and editing, methodology, software, formal analysis, and data curation. DR and YY: writing–review and editing, review, and validation. SW: writing–review and editing, conceptualization, methodology, and validation. SZ: conceptualization, supervision, project administration, and funding acquisition. KQ: data curation. All authors contributed to the article and approved the submitted version.

## Conflict of interest

The authors declare that the research was conducted in the absence of any commercial or financial relationships that could be construed as a potential conflict of interest.

## Publisher's note

All claims expressed in this article are solely those of the authors and do not necessarily represent those of their affiliated organizations, or those of the publisher, the editors and the reviewers. Any product that may be evaluated in this article, or claim that may be made by its manufacturer, is not guaranteed or endorsed by the publisher.

## Footnotes

1 Data source: https://data.sd.gov.cn/portal/index.

2 Raw data on inversion days can be downloaded from NASA's website: https://disc.gsfc.nasa.gov/datasets/M2I6NPANA_5.12.4/summary.

3 Data source: http://data.cma.cn/.
